# Long noncoding RNA SNHG6 silencing sensitized esophageal cancer cells to 5-FU via EZH2/STAT pathway

**DOI:** 10.1038/s41598-023-32607-3

**Published:** 2023-04-01

**Authors:** Ran Tan, Jia Liu, Jiang Wang, Wei Zhang, Meng He, Yueli Zhang

**Affiliations:** 1 Department of Clinical Pharmacy, Zhengzhou Central Hospital Affiliated to Zhengzhou University, Zhengzhou, China; 2 Translational Medical Center, Zhengzhou Central Hospital Affiliated to Zhengzhou University, Zhengzhou, China; 3 Department of Gastrointestinal Surgery, Zhengzhou Central Hospital Affiliated to Zhengzhou University, Zhengzhou, China

**Keywords:** Cancer, Cancer therapy, Non-coding RNAs

## Abstract

Chemotherapy was the main treatment method for esophageal cancer (EC) patients. However, chemotherapy resistance due to multiple factors is a major barrier to EC treatment. For investigating how small nucleolar RNA host gene 6 (SNHG6) affected the 5-fluorouracil (5-FU) resistance in EC as well as its possible molecular mechanism. This work conducted cell viability assay, clone formation, scratch assays together with cell apoptosis for evaluating the roles of SNHG6 and enhancer of zeste homolog 2 (EZH2, the histone-lysine N-methyltransferase). Relevant molecular mechanism was identified by RT-qPCR analysis together with Western-blot (WB) assays. Our data showed that SNHG6 expression increased in EC cells. SNHG6 promotes colony formation and migration, whereas suppresses EC cell apoptosis. SNHG6 silencing markedly promoted 5-FU-mediated suppression on KYSE150 and KYSE450 cells. Additional mechanism studies showed that SNHG6 modulating STAT3 and H3K27me3 via promoting EZH2 level. Similar to the function of SNHG6, abnormal expression of EZH2 promotes the malignancy of EC and intensifies its resistance to 5-FU. In addition, overexpression of EZH2 abolished the role of SNHG6 silencing in 5-FU sensitivity in EC cells. SNHG6 overexpression promoted malignancy of EC and increased EC cell resistance to 5-FU. Besides, further molecular mechanism studies provided a novel regulatory pathways that SNHG6 knockdown promoted EC cell sensitivity to 5-FU by modulating STAT3 and H3K27me3 via promoting EZH2 expression.

## Introduction

Esophageal cancer (EC) ranks 6th place among malignancy cancers globally, characterized by difficult to diagnose and poor prognosis^1,2^. In China, most patients lose the chance of surgery when they are first diagnosed because the early clinical symptoms of EC are difficult to detect^3^. Therefore, drug chemotherapy and radiotherapy have become a major therapeutic method to manage advanced EC cases^4^. In recent years, continuous renewal of chemotherapy has played a crucial role in improving the cure rate of EC patients. However, chemotherapy resistance due to multiple factors is a major barrier to EC treatment^5^. Therefore, it is very important to find new biomarkers or therapeutic regimens to improve the sensitivity of chemotherapy drugs for the treatment of EC.

Recently, accumulating evidence has shown that long noncoding RNA (lncRNA) do not encode proteins but participate in regulating numerous cellular events^6,7^. In particular, abnormal expression of lncRNA has an important effect on cancer genesis and progression, which functions to regulate cell growth, migration, apoptosis or other processes^8–10^. In recent years, small nucleolar RNA host gene 6 (SNHG6) shows abnormal expression within diverse tumors like EC^11^, colorectal cancer (CRC)^12^, cervical cancer (CC)^13^, and gastric cancer (GC)^14^. Abnormal expression of SNHG6 can accelerate tumor progression by promoting cell proliferation and invasion, and SNHG6 is considered to be the promising novel biomarker used to diagnose tumor^15–18^. Recently, SNHG6 is found to influence tumor malignancy through multiple pathways, such as sponge miRNAs, directly interacting with target genes or signaling pathways^11–13^.

Although new chemotherapeutic drugs and chemotherapy protocols have been rapidly updated in recent years, 5-fluorouracil (5-FU)-based chemotherapy protocols still have critical effect on treating EC^19,20^. Nonetheless, chemotherapy resistance is still the biggest factor limiting the clinical application of 5-FU in EC, and its molecular mechanism is complex and changeable^21^. Accumulating evidence has shown that lncRNA is becoming a star molecule affecting tumor chemotherapy^22,23^. Similarly, many articles verify the critical effect of lncRNA on 5-FU sensitivity in EC^24–26^. In addition, recent studies have found that abnormal expression of SNHG6 increases GC resistance to cisplatin. As revealed by one study, knockdown of SNHG6 inhibits GC resistance to cisplatin via miR-325-3p/GITR axis^27^. Another study found that SNHG6 showed interaction with miR-1297 for enhancing GC resistance to cisplatin via mBCL-2^14^.

In our prior work, SNHG6 was up-regulated within EC, which was tightly associated with EC malignancy^17^. However, whether SNHG6 can regulate 5-FU sensitivity of EC cells is still unknown. The present work will investigate how SNHG6 affected enhancing 5-FU resistance of EC and reveal its possible molecular mechanism.

## Methods and materials

### Cell culture

Healthy esophageal epithelial Het-1A cells and EC cells (KYSE150, KYSE450) were acquired from the Shanghai Cell Bank of the Chinese Academy of Sciences. All the cells were cultivated with RPMI-1640 medium, and the medium contained 10% fetal bovine serum (FBS), as well as 1% antibiotics penicillin/streptomycin. All cell lines were kept under 37 °C and 5% CO_2_ conditions. KYSE150 cell treated with 10 μM EZH2 inhibitor GSK126 (GIpBio, USA) for 72 h. Next, the concentration gradient method was used to construct 5-FU resistant EC cell lines (KYSE150/5-FU). In brief, the cells were initially incubated in medium without 5-FU for 24 h. Then, 5 µM of 5-FU medium was replaced for induction at 37 °C for 48 h. The surviving cells were transferred to a 5-FU-free medium prior to the next 5-FU treatment. After being cells to adapt to, continue to rise 25–50% of 5-FU drug concentration, repeat the above process, until 5-FU drug concentration increased to 80 µM. the concentration 40 µM of 5-FU that could stabilize the drug resistance of KYSE150 cells was selected for subsequent analysis.

### Cell transfection

Lipofectamine 3000 (Invitrogen) was utilized in cell transfection following specific instructions. Small interfering RNAs (all si-RNAs were provided by GenePharma, Shanghai, China) below were transfected into cells, including negative control (si-NC, 5′-UUCUCCGAACGUGUCACGUTT-3′); two siRNAs (siRNA1, 5′-GCAGUUUACUGAGUCAUUACU-3′ and siRNA2, 5′-UCGAAUAUGUUCAAAACAGGU-3′) were designed for silencing SNHG6, one siRNA was designed for silencing EZH2 (si‑EZH2, 5′‑AAGACTCTGAATGCAGTTGCT‑3′). Shanghai GeneChem Co., Ltd was responsible for designing and preparing pcDNA3.1 EZH2 overexpression vector (pcDNA-EZH2) and pcDNA3.1 empty vector (pcDNA‑NC).

### RT-qPCR assay

This work utilized TRIzol® (Invitrogen; Thermo Fisher Scientific, Inc.) for extracting total RNA, which was later prepared to cDNA through reverse transcription with RevertAid First Strand cDNA Synthesis Kit (Thermo Fisher Scientific). This work applied ABI 7500 RT-qPCR system in PCR with a 20 µl qPCR reaction mixture. The 2^−ΔΔCt^ approach was utilized for calculating relative gene expression, with GAPDH being the endogenous reference. Primer sequences of SNHG6, forward: 5′-ATACTTCTGCTTCGTTACCT-3′, reverse: 5′-CTCATTTTCATCATTTGCT-3′; GAPDH: 5′-GGGAGCCAAAAGGGTCAT-3′, reverse: 5′-GAGTCCTTCCACGATACCAA-3′; EZH2, forward: 5′-AGGACGGCTCCTCTAACCAT-3′, reverse: 5′-CTTGGTGTTGCACTGTGCTT-3′.

### Cell viability assay

This work utilized Cell Counting Kit-8 (CCK-8; Beyotime Institute of Biotechnology) to determine EC cell proliferation. In brief, we inoculated 5 × 10^3^ EC cells/100 µl medium in 96-well plates, and freshly prepared mediums that contained diverse 5-FU doses (0, 10, 20, 40, 60 or 80 µM) were used to replace the original medium. After 48 h incubation under 37 °C, every well was added with CCK-8 solution (10 µl) to incubate for another 2 h period. Then, absorbance (OD) value was measured at 450 nm. We used the formula below to determine cell viability, cell viability (%) = (mean OD in experimental group − mean OD in blank control group)/(mean OD in control group − mean OD in blank control group) × 100%.

### Clone formation assay

In colony forming assay, 5 × 10^2^ transfected EC cells were inoculated into the 12-well plates and cultivated them for 10 days. Then, cells were immobilized for 20 min using 4% paraformaldehyde (PFA), stained using 0.1% crystal violet (Sigma-Aldrich; Merck KGaA) for 20 min. Finally, colony number was calculated and took photographs.

### Scratch assays

Scratch assays were conducted to analyze cell migration. After transfection, we inoculated cells into 6-well plates and incubation overnight. Thereafter, this work used the 10 μl tip for making scratches on the monolayer cell. At 0/24 h, we detected wound closure and took photos.

### Cell apoptosis assay

Cells were processed using an AnnexinV/PI double staining kit for cell apoptosis assay. In Brief, transfected cells were collected and rinsed using PBS. Later, binding buffer was utilized to resuspend cells, then 15 min incubation using Annexin V-FITC as well as another 5 min incubation using PI, respectively were conducted. Flow cytometry was used for cell apoptosis assay (Beckman Coulter, Inc.).

### Western blotting (WB) analysis

This study utilized RIPA buffer (Beyotime Institute of Biotechnology) that contained protease K inhibitor for extracting total proteins, which were then separated through 12% SDS-PAGE, followed by transfer onto PVDF membrane. Subsequently, membrane was immersed within 5% defatted milk under ambient temperature for a 2 h, followed by overnight incubation under 4 °C using corresponding primary antibodies: EZH2 (1:500, ab191080; Abcam, Cambridge, MA, USA), p-STAT3 (1:2000, ab76315; Abcam, Cambridge, MA, USA), STAT3 (1:1000, ab68153; Abcam, Cambridge, MA, USA), Mcl-1 (1:1000, ab32087; Abcam, Cambridge, MA, USA) and p-Bcl-2 (1:1000, ab218123; Abcam, Cambridge, MA, USA), H3K27me3 (1:1000, ab6002; Abcam, Cambridge, MA, USA) with GAPDH being the loading reference. Then, the membrane was subject to secondary antibody incubation (1:5000, ab96899; Abcam, Cambridge, MA, USA). Protein band visualization was performed using Chemidoc EQ system (Bio-Rad Laboratories, Inc.).

### Statistical analysis

Each assay was conducted in triplicate. GraphPad Prism 8.0 was employed for statistical analysis using Student’s t-test. Experimental data are shown as the mean ± standard deviation. P < 0.05 stood for statistical significance.

### Ethics approval and consent to participate

The study was approved by the Medical Ethics Committee of the Zhengzhou Central Hospital.

## Results

### LncRNA SNHG6 inhibits EC cells sensitivity to 5-FU

**Figure 1:**
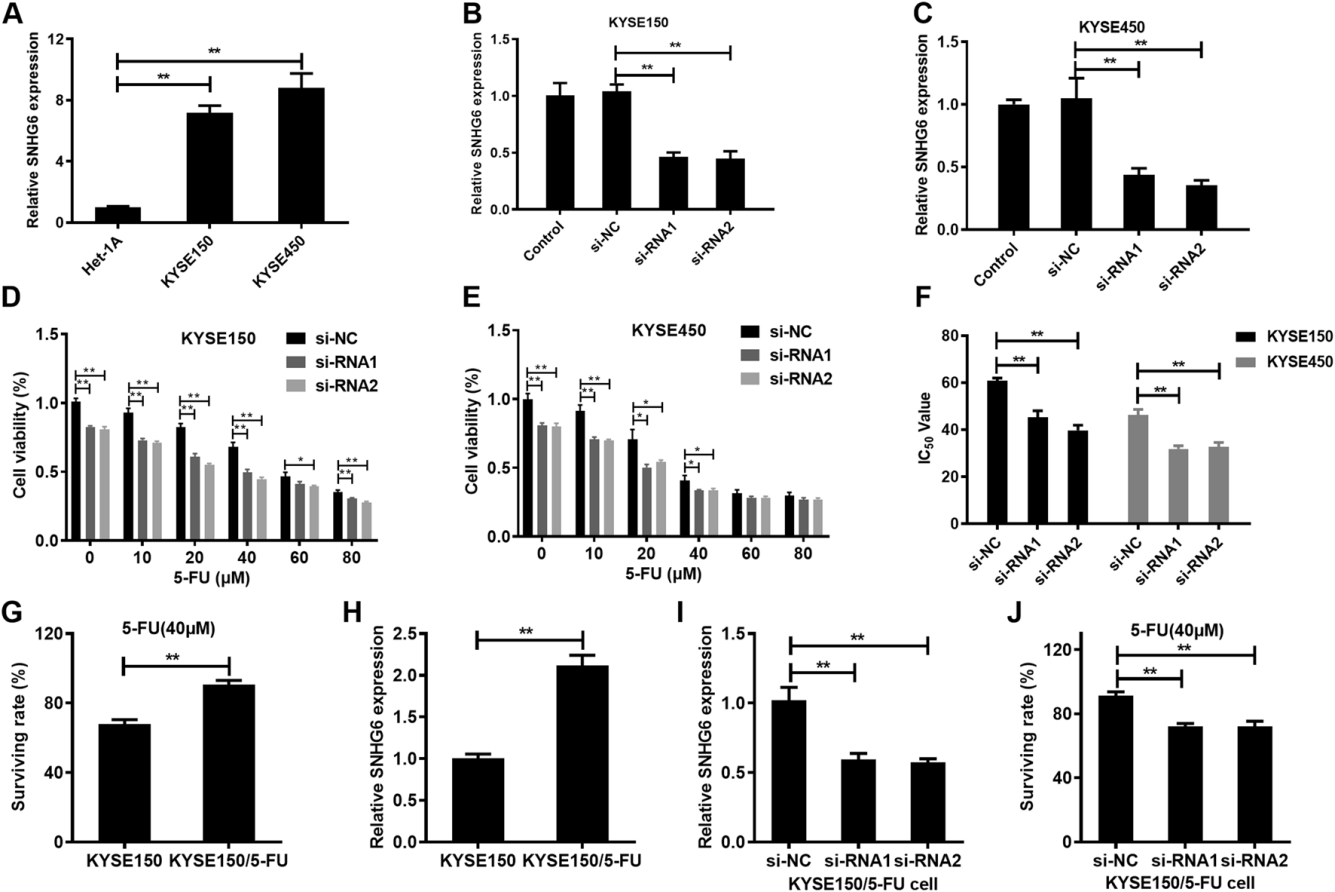
LncRNA SNHG6 inhibits EC cells sensitivity to 5-FU. (**A**) SNHG6 levels in EC cells and Het-1A cells. (**B**, **C**) Transfection of si-RNAs reduces SNHG6 expression in EC cells. (**D**, **E**) Transfection of si-RNAs reduces EC cells viability at varying concentrations of 5-FU. (**F**) Transfection of si-RNAs reduces the IC_50_ of 5-FU to EC cells. (**G**) Surviving rate of KYSE150 cell and KYSE150/5-FU cell. (**H**) SNHG6 levels in KYSE150 cell and KYSE150/5-FU cell. (**I**) Transfection of si-RNAs reduces SNHG6 expression in KYSE150/5-FU cell. (**J**) Transfection of si-RNAs reduces the surviving rate of KYSE150/5-FU cell. *P < 0.05; **P < 0.01 (Two-way Student’s t-test).

This work first conducted RT-qPCR to detect SNHG6 levels within EC cells. SNHG6 expression markedly increased within KYSE150 and KYSE450 cells relative to in Het-1A cell (Fig. 1A). To identify the effect of SNHG6 on 5-FU sensitivity to EC cells, two si-RNAs were designed to knock down SNHG6 expression. SNHG6 expression was significantly reduced with si-RNAs transfection in KYSE150 and KYSE450 cells (Fig. 1B,C). To explore the effect of knock down SNHG6 on EC cell sensitivity to 5-FU, KYSE150 and KYSE450 cells transfected with si-RNAs or si-NC were treated with 50 µM 5-FU. Knock down SNHG6 markedly promoted 5-FU-mediated suppression on KYSE150 and KYSE450 cells (Fig. 1D,E). Additionally, this work also examined how SNHG6 affected IC_50_ of 5-FU to KYSE150 and KYSE450 cells. The results showed that knock down SNHG6 significantly reduced the IC_50_ of 5-FU to KYSE150 and KYSE50 cells (Fig. 1F).

Then, we explored the effect of SNHG6 on 5-FU sensitivity in drug-resistant cell lines. We first used CCK-8 assay to compare the surviving rate of KYSE150 cell line and KYSE150/5-FU cell line at a 5-FU concentration of 40 µM, and found that the surviving rate of KYSE150/5-FU cell line was significantly increased relative to that of KYSE150 cell line (Fig. 1G). Then, the expression of SNHG6 was detected by qRT-PCR, and found that the expression of SNHG6 of KYSE150/5-FU cell line was significantly increased relative to that of KYSE150 cell line (Fig. 1H). We further evaluated the effect of SNHG6 knockdown on the sensitivity of KYSE150/5-FU cell line to 5-FU. SNHG6 expression was significantly reduced with si-RNAs transfection in KYSE150/5-FU cell (Fig. 1I). The surviving rate of si-RNA1 and si-RNA2 group were significantly increased relative to that of si-NC group (Fig. 1J). This results suggested that knocking down SNHG6 could increase the sensitivity of KYSE150/5-FU cell to 5-FU.

### SNHG6 promotes colony formation and migration, while suppresses apoptosis of EC cells

**Figure 2:**
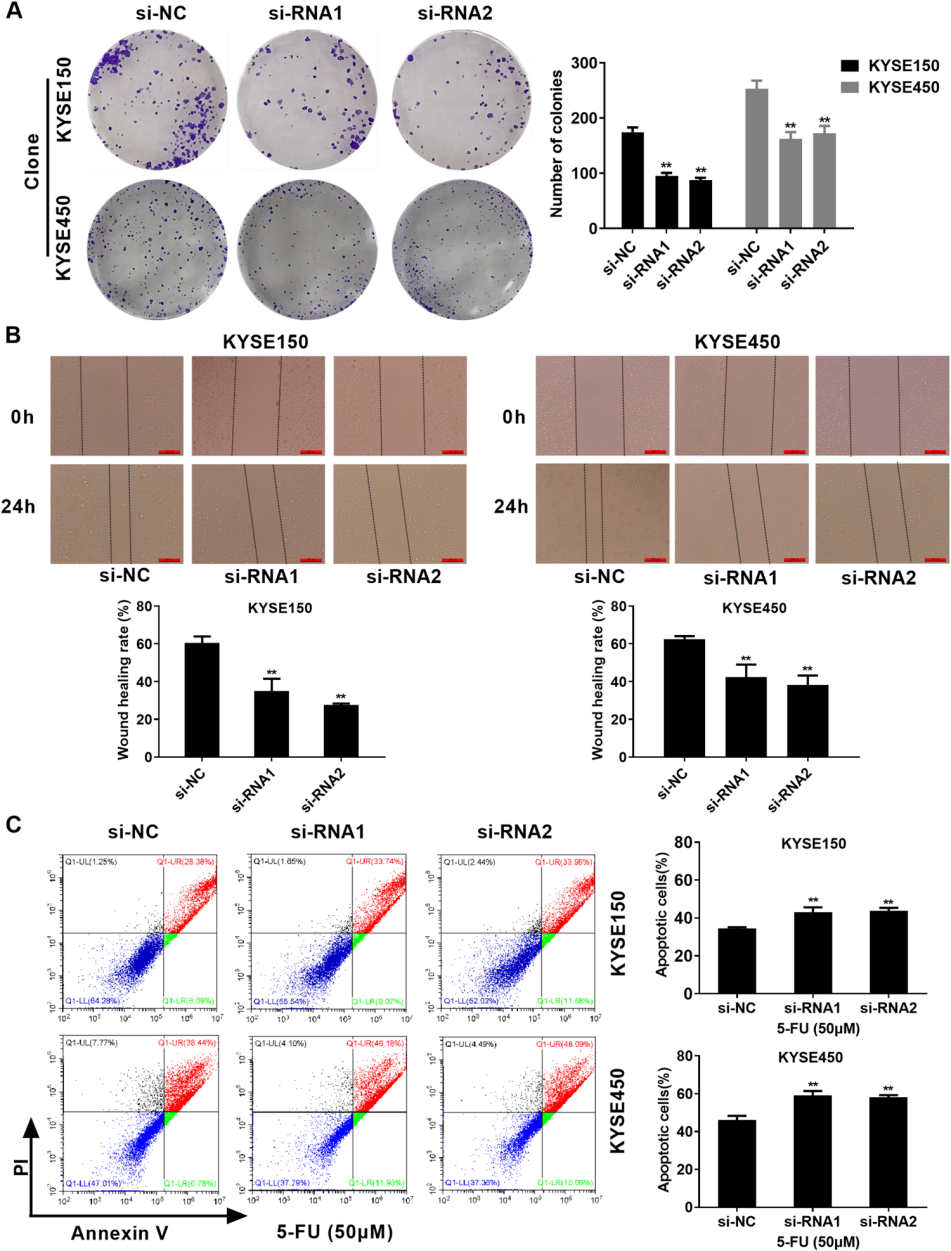
SNHG6 promotes colony formation and migration, while suppresses apoptosis of EC cells. (**A**) Transfection of si-RNAs inhibits colony formation of EC cells. (**B**) Transfection of si-RNAs inhibits migration of EC cells. (**C**) Transfection of si-RNAs promotes apoptosis of EC cells. **P < 0.01, compared with si-NC group (Two-way Student’s t-test).

The present work further evaluated how SNHG6 knockdown affected colony formation, migration and apoptosis of KYSE150 and KYSE450 cells. Colony formation assay found that knock down SNHG6 significantly inhibited clonogenic ability of KYSE150 and KYSE450 cells (Fig. 2A). As revealed by wound healing assay, knock down SNHG6 significantly suppressed wound healing rate of KYSE150 and KYSE450 cells (Fig. 2B). In addition, cell count analysis demonstrated that knock down SNHG6 remarkably promoted apoptosis of KYSE150 and KYSE450 cells (Fig. 2C). Based on the above findings, SNHG6 was possibly related to regulating killing ability of 5-FU on EC cells.

### SNHG6 regulates the EZH2/STAT3 pathway

**Figure 3:**
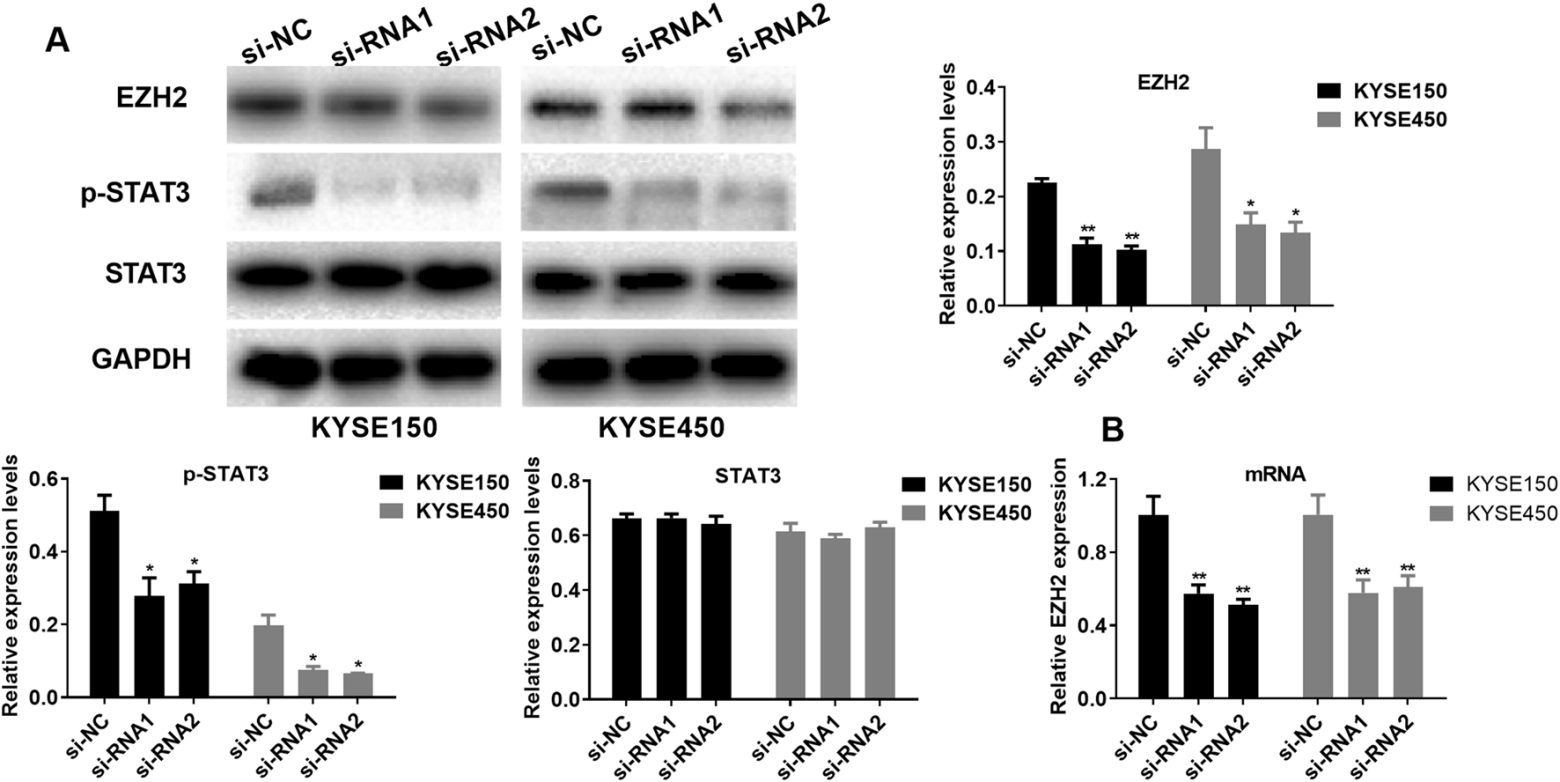
SNHG6 regulates the EZH2/STAT3 pathway in EC cells. (**A**) Role of si-RNAs transfection in protein levels of EZH2, p-STAT3 and STAT3. (**B**) Role of si-RNAs transfection in mRNA levels of EZH2. *P < 0.05; **P < 0.01, compared with si-NC group (Two-way Student’s t-test).

For better exploring how SNHG6 promoted EC cells malignancy, protein expression of EZH2 and downstream pathway was detected by western blotting. As a result, transfection of si-RNAs markedly suppressed EZH2 protein level with KYSE150 and KYSE450 cells. Meanwhile, transfection of si-RNAs remarkably suppressed p-STAT3 protein level, whereas the transfection has no significant effect on protein expression of STAT3 with KYSE150 and KYSE450 cells (Fig. 3A). In addition, transfection of si-RNAs significantly inhibited the mRNA expression of EZH2 (Fig. 3B). These results indicated that SNHG6 may regulate EC cells malignancy via promoting phosphorylation of STAT3.

### EZH2 promotes growth of EC cells and inhibits EC cells sensitivity to 5-FU

**Figure 4:**
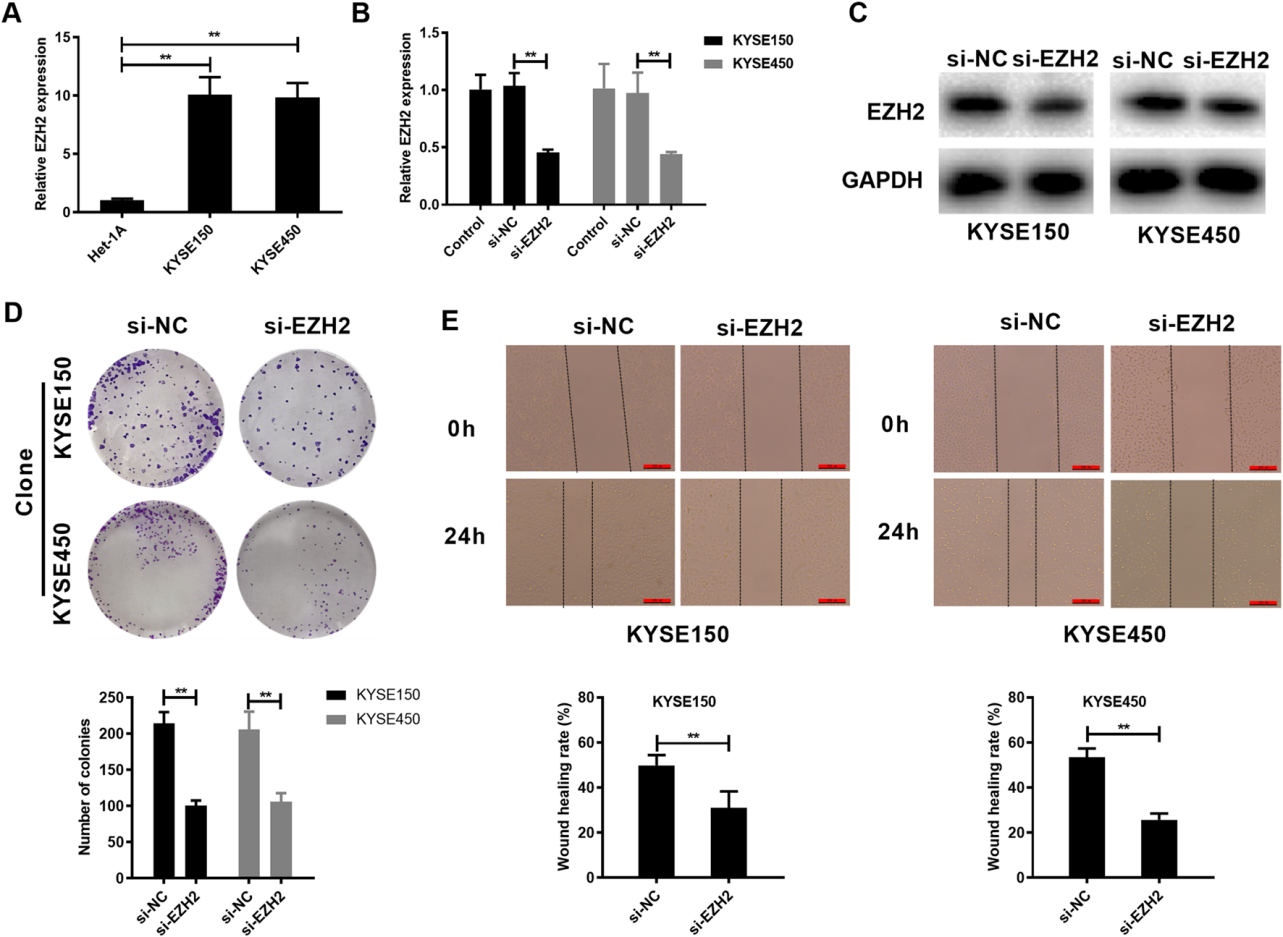
EZH2 promotes colony formation and invasion in EC cells. (**A**) EZH2 levels within EC cells and Het-1A cells. (**B**, **C**). Transfection of si-EZH2 reduces EZH2 expression in mRNA and protein levels. (**D**) Transfection of si-EZH2 inhibits colony formation of EC cells. (**E**) Transfection of si-EZH2 inhibits migration of EC cells. **P < 0.01 (Two-way Student’s t-test).

**Figure 5:**
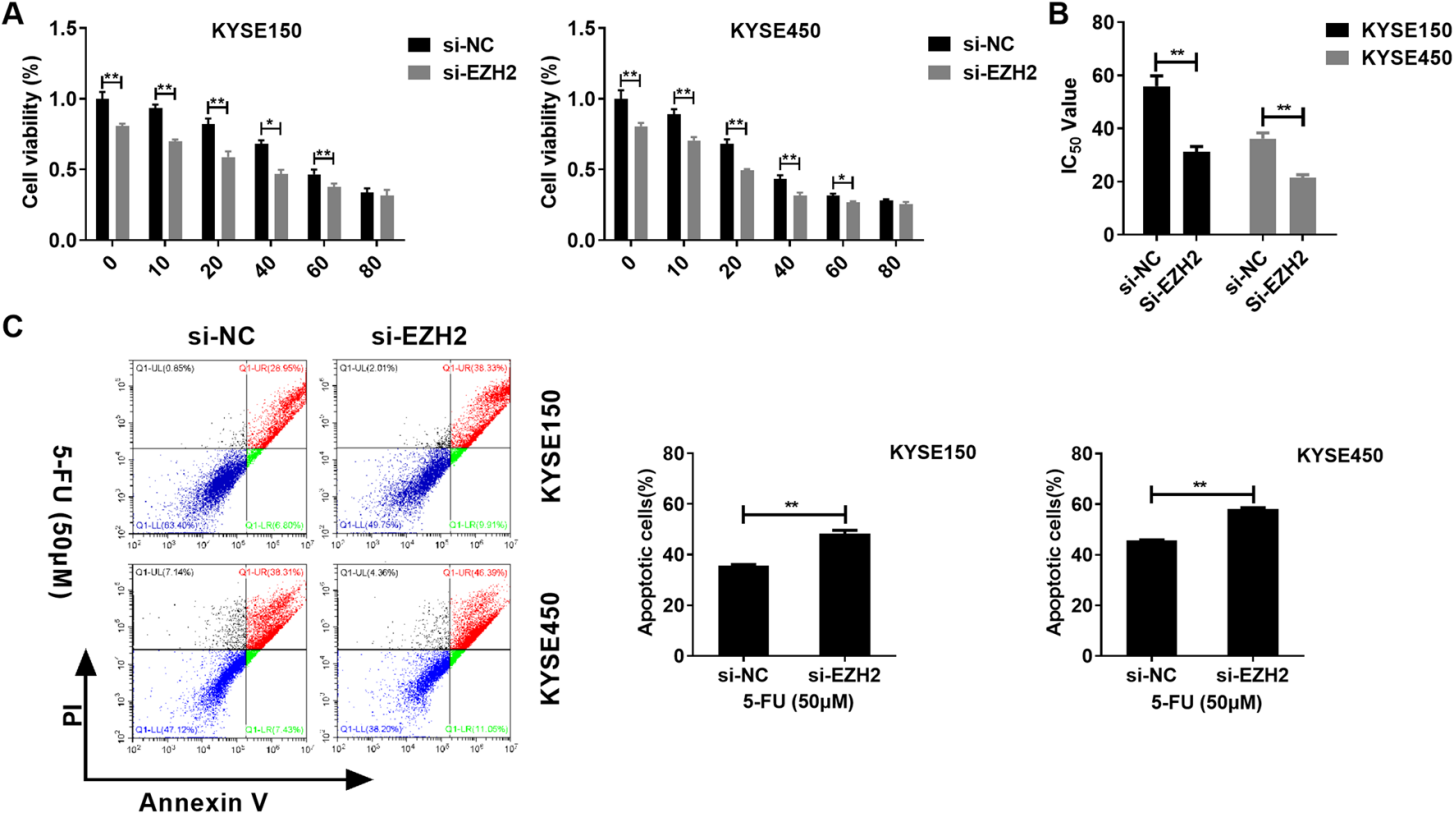
EZH2 inhibits EC cells sensitivity to 5-FU. (**A**) Transfection of si-EZH2 reduces EC cells viability at varying concentrations of 5-FU. (**B**) Transfection of si-EZH2 reduces the IC_50_ of 5-FU to EC cells. (**C**) Transfection of si-EZH2 promotes apoptosis of EC cells. *P < 0.05; **P < 0.01 (Two-way Student’s t-test).

This section examined EZH2 level within cells, as a result, EZH2 expression significantly increased within KYSE150 and KYSE450 cells relative to in Het-1A cell (Fig. 4A). Similarly, si-RNA was designed to silencing EZH2. si-EZH2 transfection significantly inhibited the EZH2 protein and mRNA levels within KYSE150 and KYSE450 cells (Fig. 4B,C). This work also evaluated the effect of EZH2 knock down on colony formation and migration of EC cells. Colony formation assay found that transfection of si-EZH2 significantly inhibited clonogenic ability of EC cells (Fig. 4D). Wound healing assay shown that transfection of si-EZH2 significantly suppressed wound healing rate of EC cells (Fig. 4E). Then, we evaluated the effect of knock down EZH2 on EC cell sensitivity to 5-FU. Knock down EZH2 markedly promoted 5-FU-mediated suppression on KYSE150 and KYSE450 cells and reduced the IC_50_ of 5-FU to KYSE150 and KYSE450 cells (Fig. 5A,B). In addition, knock down EZH2 remarkably promoted apoptosis of KYSE150 and KYSE450 cells (Fig. 5C).

### EZH2 inhibits EC cell apoptosis by activating the STAT3 pathway

**Figure 6:**
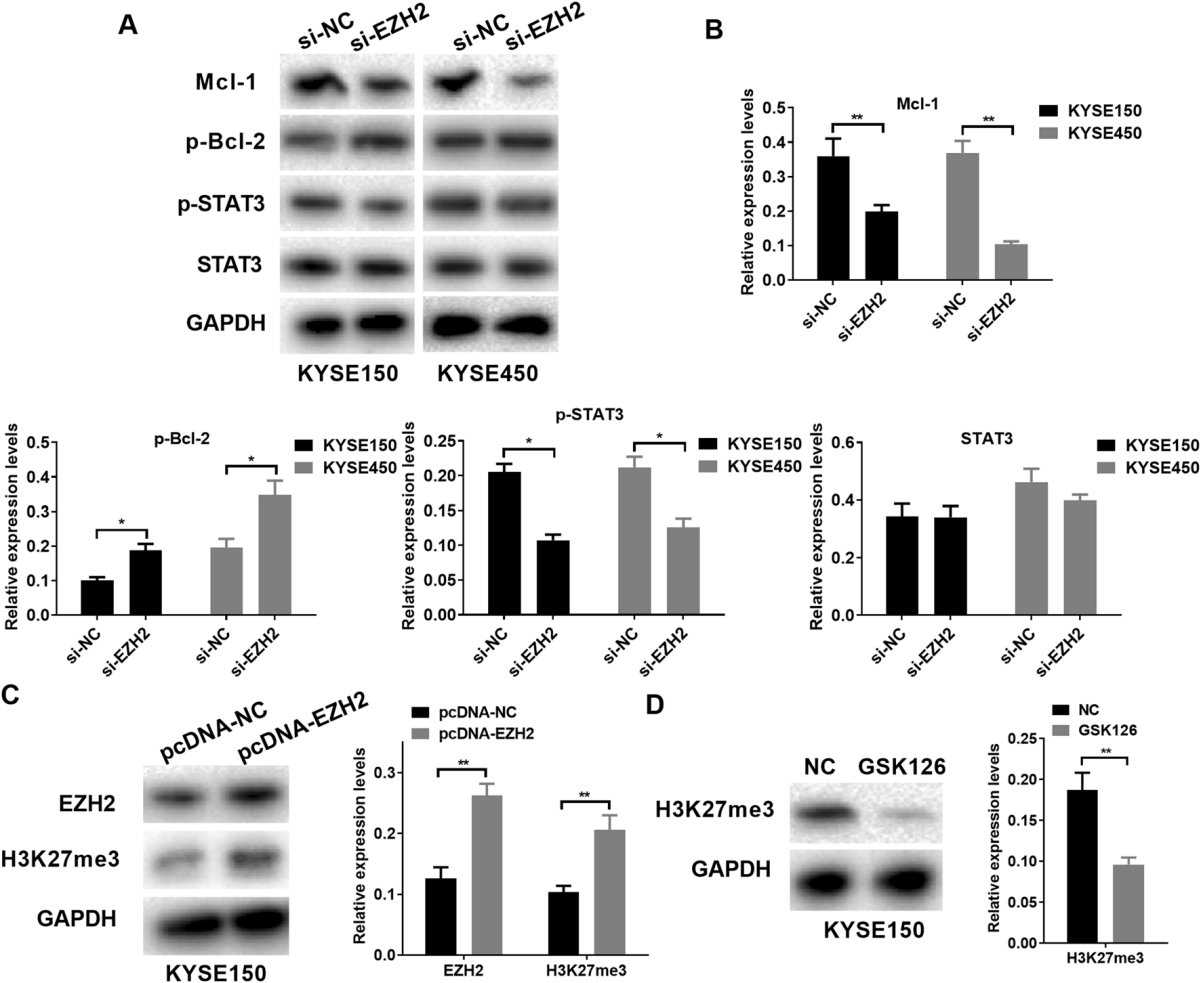
EZH2 inhibits EC cell apoptosis by activating the STAT3 pathway. (**A**, **B**) Role of si-EZH2 transfection in protein levels of Mcl-1, p-Bcl-2, p-STAT3 and STAT3. (**C**) Role of pcDNA-EZH2 transfection in protein levels of EZH2 and H3K27me3. (**D**) KYSE150 cell treated with GSK126 significantly inhibited the expression of H3K27me3. *P < 0.05; **P < 0.01 (Two-way Student’s t-test).

For exploring EZH2 mechanism in promoting EC cell sensitivity to 5-FU, this study also examined protein expression associated with the STAT3 pathway and apoptosis. As a result, transfection of si-EZH2 markedly suppressed Mcl-1 protein level, while increased p-Bcl-2 with KYSE150 and KYSE450 cells. Meanwhile, transfection of si-EZH2 remarkably suppressed p-STAT3 protein level, while the transfection has no significant effect on protein expression of STAT3 with KYSE150 and KYSE450 cells (Fig. 6A,B). These results indicated that EZH2 may regulate EC cells apoptosis by activating the STAT3 pathway. In order to further investigate the changes of histone methylation, we first detected the effects of overexpression of EZH2 on the EZH2 and H3K27me3 expression, and found that EZH2 overexpression significantly increased the protein expression of EZH2 and H3K27me3 in KYSE150 cell (Fig. 6C). In addition, KYSE150 cell treated with GSK126 significantly inhibited the expression of H3K27me3 in KYSE150 cell (Fig. 6D).

### Overexpression of EZH2 abolished the role of SNHG6 silencing on EC cells sensitivity to 5-FU

**Figure 7:**
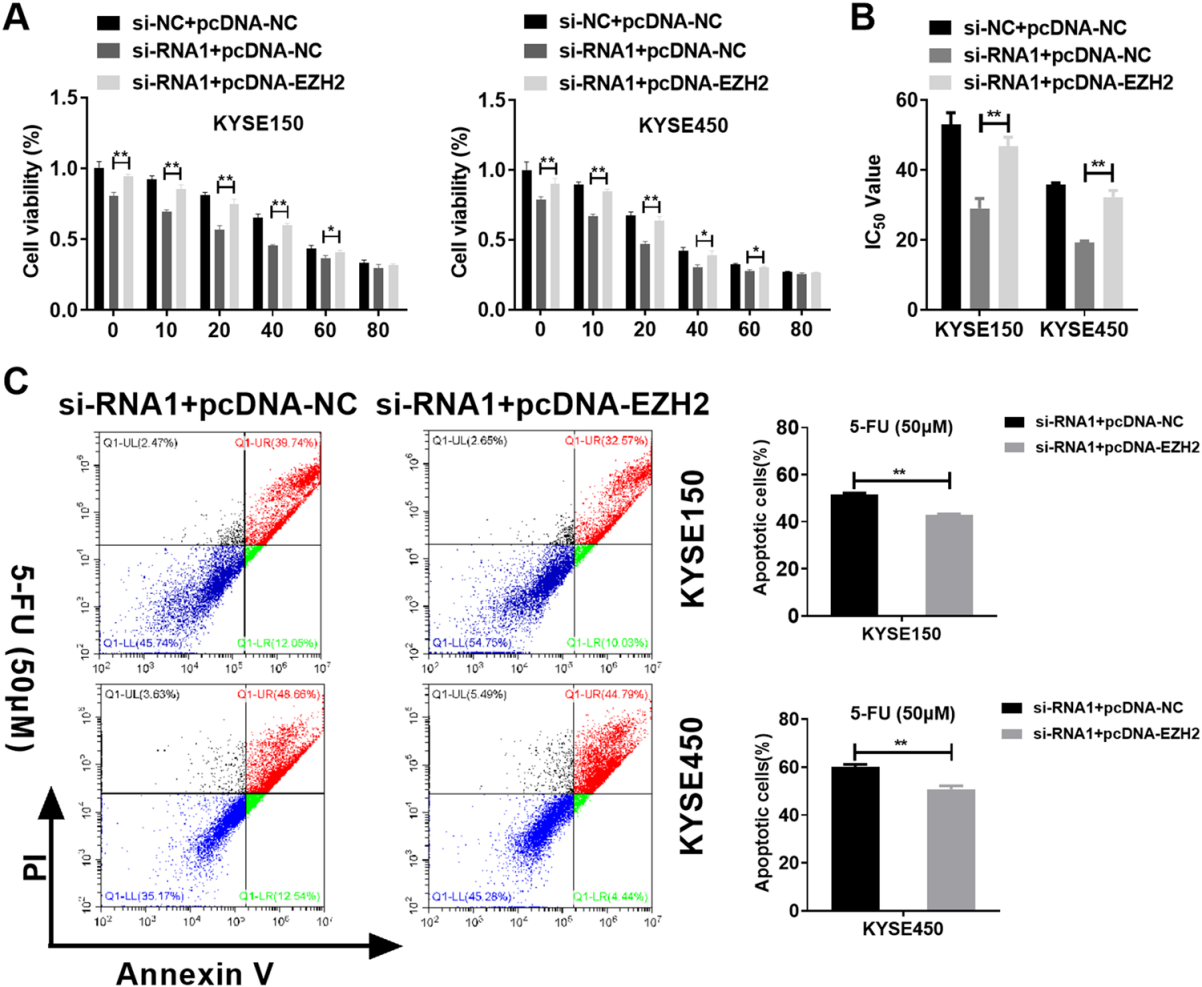
Overexpression of EZH2 abolished the role of SNHG6 silencing on EC cells sensitivity to 5-FU. (**A**) Role of si-RNA1 and pcDNA-EZH2 co-transfection in cells viability of EC cells. (**B**) Role of si-RNA1 and pcDNA-EZH2 co-transfection in IC_50_ of 5-FU to EC cells. (**C**) Role of si-RNA1 and pcDNA-EZH2 co-transfection in apoptosis of EC cells. *P < 0.05; **P < 0.01 (Two-way Student’s t-test).

Our previous studies have confirmed the effects of SNHG6 and EZH2 on 5-FU sensitivity to EC cells, and SNHG6 could regulation of EZH2 level. Therefore, we would like to observe whether SNHG6 can affect 5-FU sensitivity to EC cells by modulating EZH2 level. To test this hypothesis, pcDNA-EZH2 was co-transfected with si-RNA1 in EC cells. Compared with si-RNA1 + pcDNA-NC group, co-transfection of si-RNA1 + pcDNA-EZH2 significantly reduced 5-FU sensitivity to EC cells and enhanced the IC_50_ of 5-FU to KYSE150 and KYSE450 cells (Fig. 7A,B). Next, we monitored the effect of co-transfection of si-RNA1 and pcDNA-EZH2 on apoptosis EC cells. Compared with si-RNA1 + pcDNA-NC group, co-transfection of si-RNA1 + pcDNA-EZH2 inhibited apoptosis of EC cells (Fig. 7C). As a result, EZH2 up-regulation partly abolished SNHG6 silencing’s function in 5-FU sensitivity to EC cells.

## Discussion

Recent studies have shown that lncRNAs have become important biomarkers for cancer and may be potential therapeutic targets^23,28,29^. According to our previous studies, SNHG6 showed marked up-regulation within EC, which might be a biomarker for the diagnosis of EC^17^. However, studies on the effect of SNHG6 on 5-Fu sensitivity in EC cells are rarely reported. This study confirmed that SNHG6 inhibits 5-FU sensitivity in EC cells. And revealed the molecular mechanism that SNHG6 knockdown promoted EC cell sensitivity to 5-FU by regulating EZH2/STAT3 pathway.

SNHG6 has been previously suggested to be the candidate new anti-tumor therapeutic target. SNHG6 was found to reduce the PTX sensitivity of prostate cancer (PCa) cells via the sponge of miR-186, which indicated that SNHG6 was the possible anti-PCa therapeutic target^30^. Other research also confirmed that SNHG6 silencing increases cisplatin sensitivity in GC cells^14,27^. We also confirmed that SNHG6 promotes EC malignancy, and knockdown of SNHG6 enhanced 5-FU sensitivity in EC cells, but weakened drug resistance. Related studies also confirmed the influence of lcnRNAs on 5-FU sensitivity in EC cells. For example, in one study, lncRNA HOTAIR inhibits 5-FU sensitivity in EC cells by mediating MTHFR methylation^26^. Another study found that LINC00261 promoted the 5-FU chemosensitization through regulating DPYD suppression dependent on methylation within EC^31^. And another study also revealed that LINC01419 decreased 5-FU sensitivity in ESCC cells by mediating GSTP1 methylation^32^. In addition, SNHG6 can enhance chemoresistance of 5-FU by the ULK1-mediated autophagy through the sponge of miR-26a-5p within CRC cells^33^. These suggested that SNHG6 induce chemoresistance of 5-FU in EC.

As revealed that SNHG6 regulates the malignancy of tumors through multiple pathways^11–13^. According to our results, SNHG6 and EZH2 synergistically promoted EC malignancy and SNHG6 modulated EZH2 level. Successive studies have found that SNHG6 can promote tumor malignancy by regulating the expression of EZH2, including in CRC^34^, GC^35^, and ovarian clear cell carcinoma^36^. Our previous research also confirmed that SNHG6 regulates EZH2 level via the sponge of miR-101-3p within EC^37^. In addition, the current study confirmed that SNHG6 and EZH2 synergistically increase chemotherapeutic resistance to 5-FU in EC. Overexpression of EZH2 abolished the role of SNHG6 silencing on 5-FU sensitivity in EC cells. A study also revealed that EZH2 enhances the chemotherapeutic resistance of 5-FU via up-regulating PUMA in colorectal cancer^38^. Another study confirmed that decreased EZH2 expression increased EC cells sensitivity to 5-FU^39^. These suggested that SNHG6 enhances the 5-FU chemotherapeutic resistance through modulating EZH2 level in EC.

To better investigate SNHG6/EZH2 role in the 5-FU sensitivity within EC, this study estimated EZH2’s impacts on downstream pathways. STAT3, which belongs to the family of cytoplasmic transcription factors of STAT, mediates multiple intracellular signaling pathways and biological processes like cell growth, differentiation, apoptosis, and angiogenesis of tumors^40,41^. STAT3 can be activated through phosphorylating the conserved serine and tyrosine residues within the C-terminal domains mediated via JAK proteins^42^. In this study, we revealed that EZH2 activates STAT3 by promoting its phosphorylation and further affecting the expression of apoptosis-related proteins. Related studies have also confirmed the effect of EZH2 on STAT3 phosphorylation^43,44^. Additionally, various articles confirmed that STAT3 pathway activation enhances chemotherapy resistance of 5-FU. Such as, one study revealed that inhibition of JAK2/STAT3 pathway reduced 5-FU resistance of gastric cancer^45^. Another study revealed that troxerutin enhances 5-FU sensitivity to GC by inhibiting Bcl-2 and STAT3/NF-κB pathways^46^. These indicated that SNHG6 promotes the 5-FU chemotherapeutic resistance through modulating EZH2 level to activate the STAT3 pathway. In addition, we found that regulation of EZH2 modulated H3K27me3 expression. Related studies have also found histone H3K27 trimethylation modulates 5-fluorouracil resistance by inhibiting pu.1 binding to the dpyd promoter^47^. This may be another mechanism by which SNHG6 regulates 5-FU resistance by modulating EZH2 and H3K27me3.

**Figure 8:**
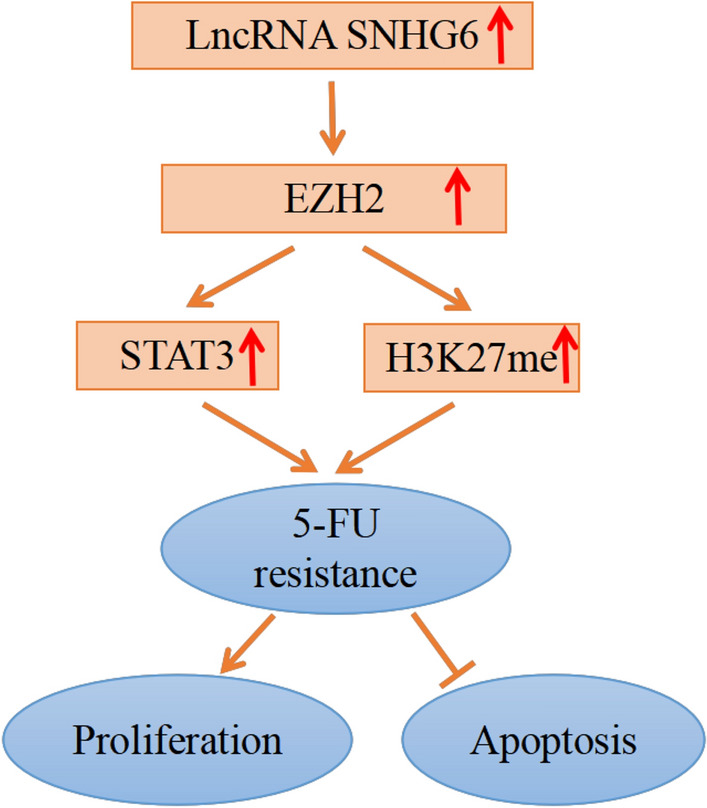
Schematic illustration of SNHG6/EZH2/STAT3 axis in regulating 5-FU resistance in EC.

In conclusion, up-regulation of SNHG6 enhanced EC malignancy grade and decreased EC cell sensitivity to 5-FU. The mechanism study revealed that SNHG6 enhanced the EC cell resistance to 5-FU by modulating STAT3 and H3K27me3 via promoting EZH2 expression (Fig. 8). This research indicated that SNHG6 is possibly the valuable biomarker used to diagnose and treat EC (Supplementary Figures).

## Supplementary Information

Supplementary Figures.

## Data Availability

The data that support the findings of this study are available from the corresponding author upon reasonable request.
