# Supplementary material for: Long noncoding RNA SNHG6 silencing sensitized esophageal cancer cells to 5-FU via EZH2/STAT pathway
Source: Sci Rep. 2023 Apr 1;13:5363. doi: 10.1038/s41598-023-32607-3 (PMC10067833; doi:10.1038/s41598-023-32607-3)
Supplement: Supplementary file 1 — Supplementary Figures. [file 41598_2023_32607_MOESM1_ESM.docx]

**Supplementary Figure for:**

**Long noncoding RNA SNHG6 silencing sensitized esophageal cancer cells to 5-FU via EZH2/STAT pathway**

**Ran Tan^1^, Jia Liu^2^, Jiang Wang^3^, Wei Zhang^1^, Meng He^1^, Yueli Zhang^1^**^🖂^

^1^Department of clinical pharmacy, Zhengzhou Central Hospital Affiliated to Zhengzhou University, Zhengzhou, China. ^2^Translational Medical Center, Zhengzhou Central Hospital Affiliated to Zhengzhou University, Zhengzhou, China. ^3^Department of Gastrointestinal Surgery, Zhengzhou Central Hospital Affiliated to Zhengzhou University, Zhengzhou, China. ^🖂^email: Zhangyueli0228@163.com


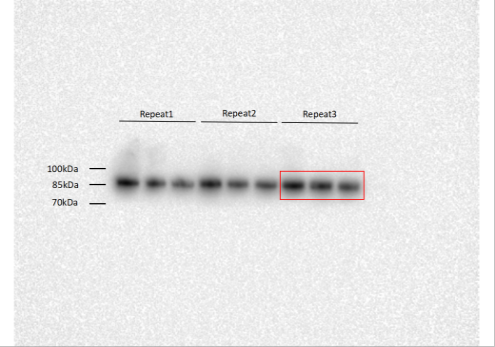


Figure3A-EZH2 (KYSE150)


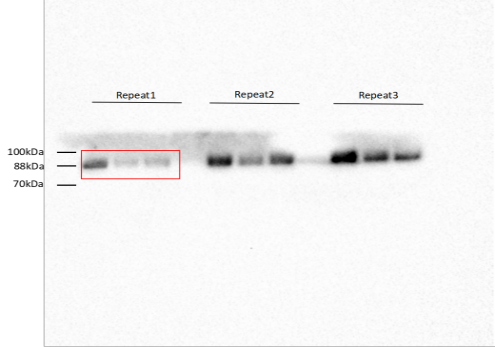


Figure3A-p-STAT3 (KYSE150)


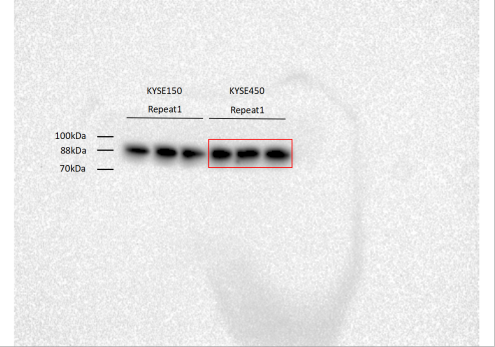


Figure3A-STAT3


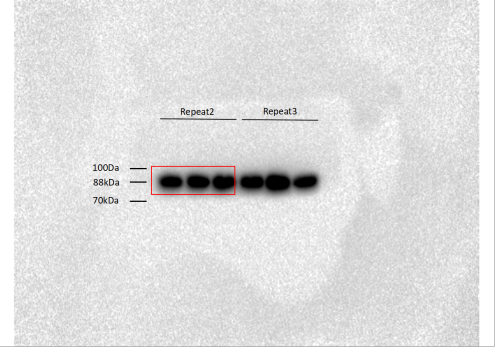


Figure3A-STAT3 (KYSE150)


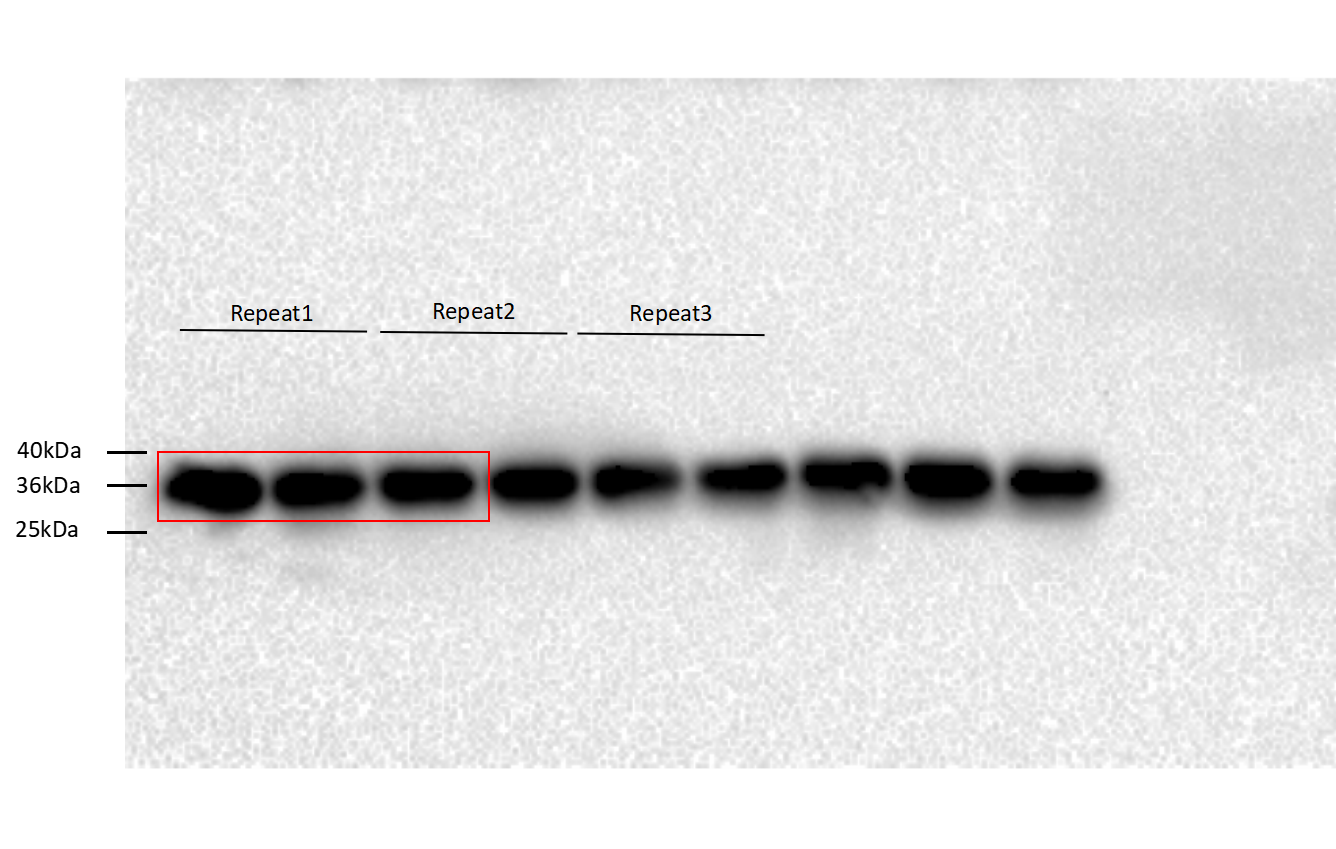


Figure3A-GAPDH (KYSE150)


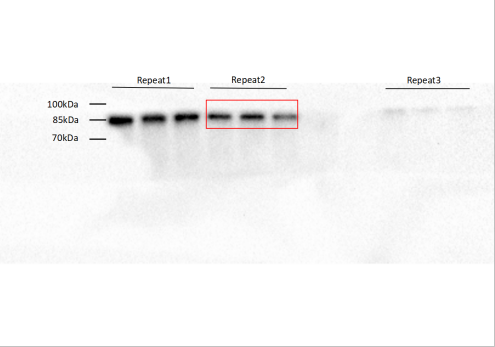


Figure3A-EZH2 (KYSE450)


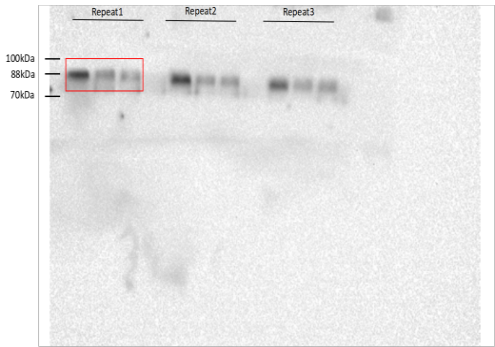


Figure3A-p-STAT3 (KYSE450)


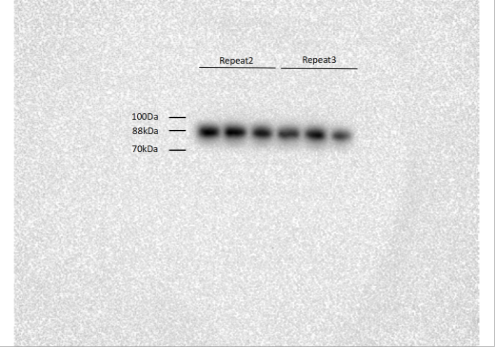


Figure3A-STAT3 (KYSE450)


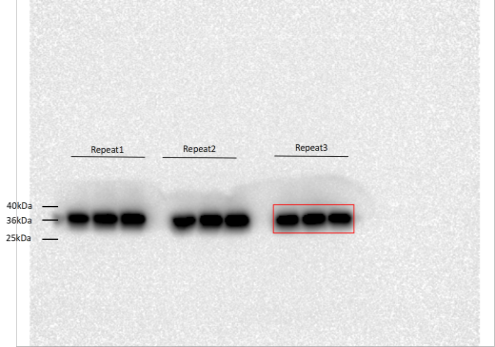


Figure3A-GAPDH (KYSE450)

Supplementary Figure 1. Unprocessed western blots. Source Data for Figure 3A.


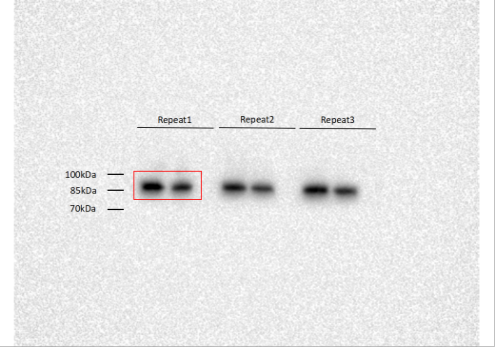


Figure4C-EZH2 (KYSE150)


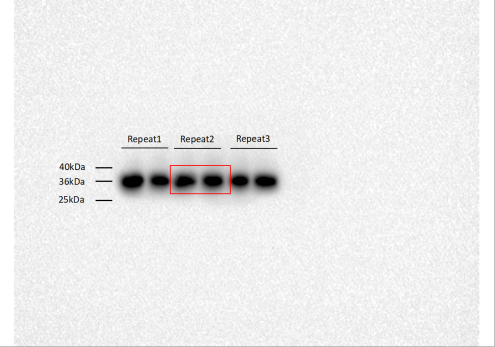


Figure4C-GAPDH (KYSE150)


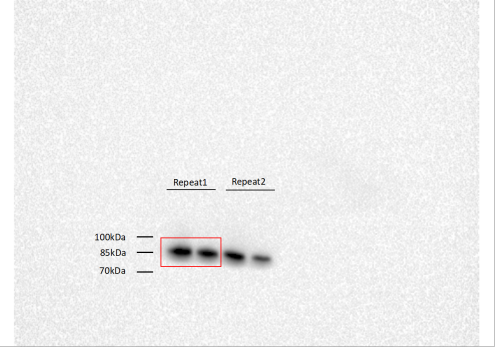


Figure4C-EZH2 (KYSE150)


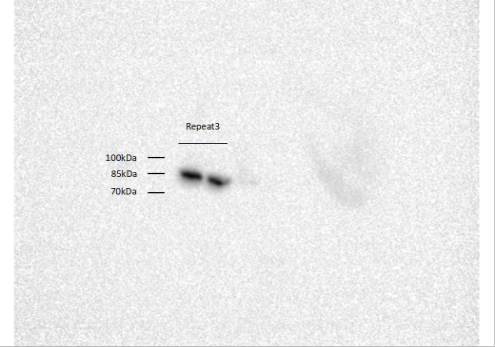


Figure4C-EZH2 (KYSE150)


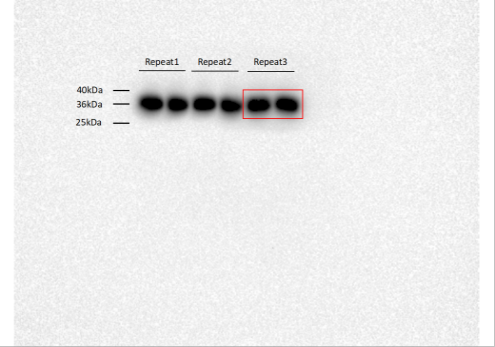


Figure4C-GAPDH (KYSE450)

Supplementary Figure 2. Unprocessed western blots. Source Data for Figure 4C.


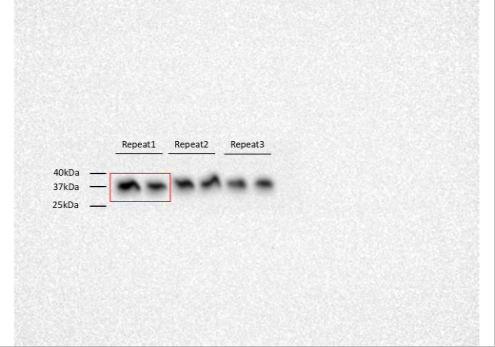


Figure6A-Mcl-1 (KYSE150)


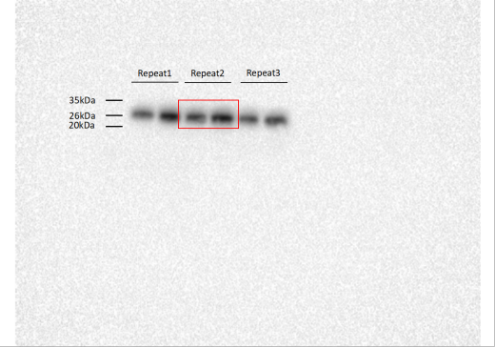


Figure6A-p-Bcl-2 (KYSE150)


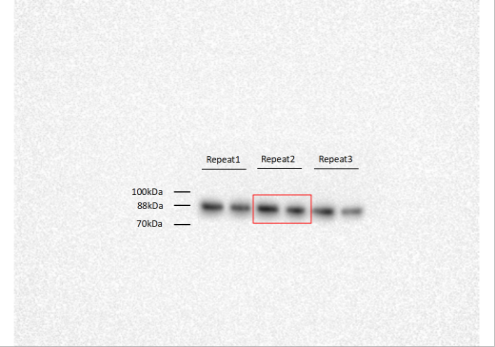


Figure6A-p-STAT3 (KYSE150)


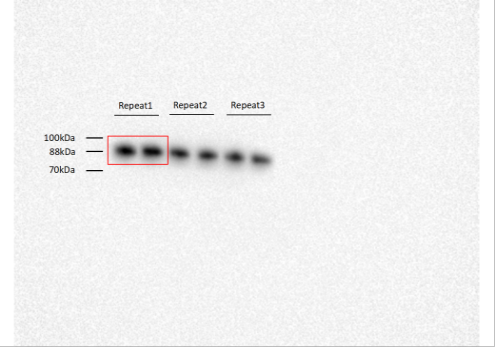


Figure6A-STAT3 (KYSE150)


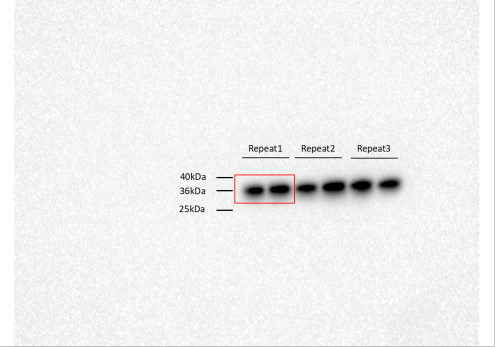


Figure6A-GAPDH (KYSE150)


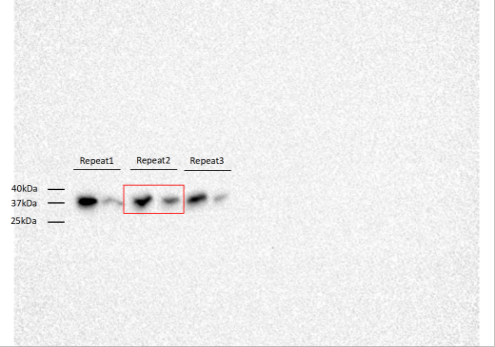


Figure6A-Mcl-1 (KYSE450)


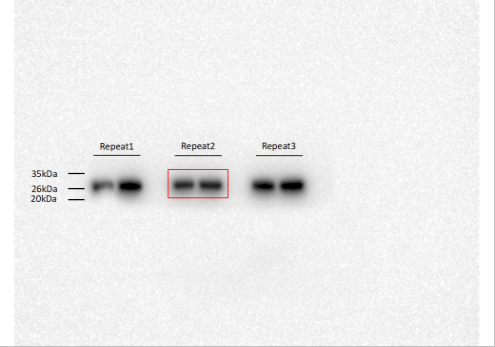


Figure6A-p-Bcl-2 (KYSE450)


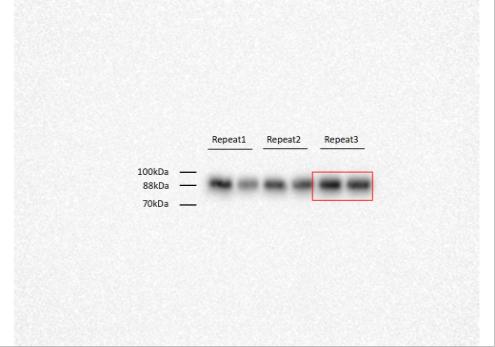


Figure6A-p-STAT3 (KYSE450)


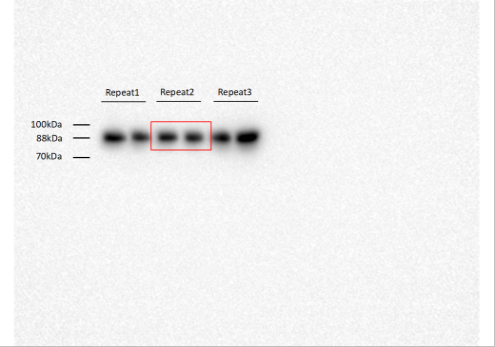


Figure6A-STAT3 (KYSE450)


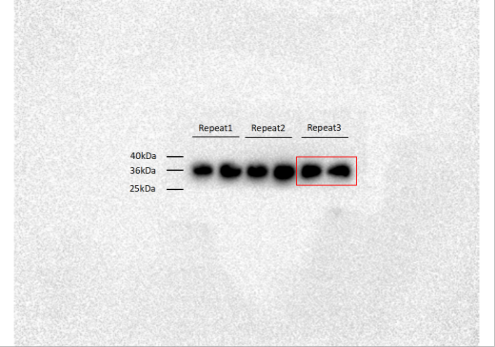


Figure6A-GAPDH (KYSE450)

Supplementary Figure 3. Unprocessed western blots. Source Data for Figure 6A.


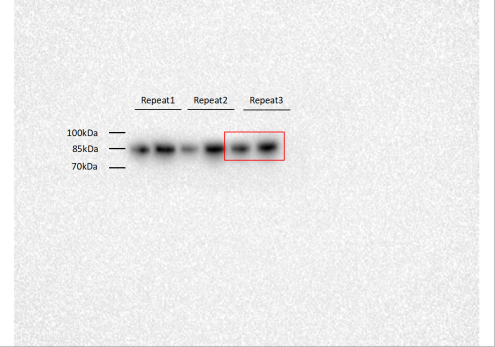


Figure6C-EZH2 (KYSE150)


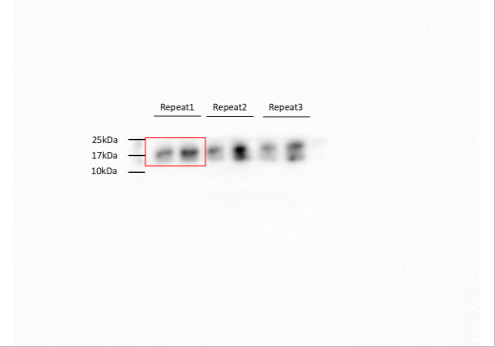


Figure6C-H3K27me3 (KYSE150)


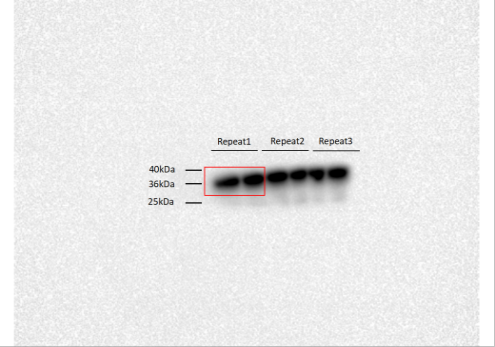


Figure6C-GAPDH (KYSE150)

Supplementary Figure 4. Unprocessed western blots. Source Data for Figure 6C.


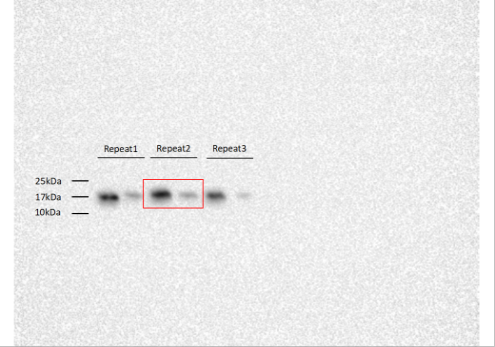


Figure6D-H3K27me3 (KYSE150)


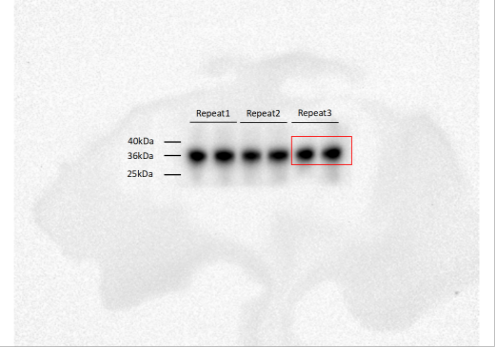


Figure6D-GAPDH (KYSE150)

Supplementary Figure 5. Unprocessed western blots. Source Data for Figure 6D.
